# A network flow approach to predict drug targets from microarray data, disease genes and interactome network - case study on prostate cancer

**DOI:** 10.1186/2043-9113-2-1

**Published:** 2012-01-13

**Authors:** Shih-Heng Yeh, Hsiang-Yuan Yeh, Von-Wun Soo

**Affiliations:** 1 Department of Computer Science, National Tsing Hua University, HsinChu 300, Taiwan; 2 Institute of Information Systems and Applications, National Tsing Hua University, HsinChu 300, Taiwan

**Keywords:** Drug target prediction, network flow, gene expression, interactome network, prostate cancer

## Abstract

**Background:**

Systematic approach for drug discovery is an emerging discipline in systems biology research area. It aims at integrating interaction data and experimental data to elucidate diseases and also raises new issues in drug discovery for cancer treatment. However, drug target discovery is still at a trial-and-error experimental stage and it is a challenging task to develop a prediction model that can systematically detect possible drug targets to deal with complex diseases.

**Methods:**

We integrate gene expression, disease genes and interaction networks to identify the effective drug targets which have a strong influence on disease genes using network flow approach. In the experiments, we adopt the microarray dataset containing 62 prostate cancer samples and 41 normal samples, 108 known prostate cancer genes and 322 approved drug targets treated in human extracted from DrugBank database to be candidate proteins as our test data. Using our method, we prioritize the candidate proteins and validate them to the known prostate cancer drug targets.

**Results:**

We successfully identify potential drug targets which are strongly related to the well known drugs for prostate cancer treatment and also discover more potential drug targets which raise the attention to biologists at present. We denote that it is hard to discover drug targets based only on differential expression changes due to the fact that those genes used to be drug targets may not always have significant expression changes. Comparing to previous methods that depend on the network topology attributes, they turn out that the genes having potential as drug targets are weakly correlated to critical points in a network. In comparison with previous methods, our results have highest mean average precision and also rank the position of the truly drug targets higher. It thereby verifies the effectiveness of our method.

**Conclusions:**

Our method does not know the real ideal routes in the disease network but it tries to find the feasible flow to give a strong influence to the disease genes through possible paths. We successfully formulate the identification of drug target prediction as a maximum flow problem on biological networks and discover potential drug targets in an accurate manner.

## Background

Cancer is extremely complex and it is viewed as a multi-stage disease caused by the accumulation of genetic alterations in tumor associated suppressor oncogenes [1]. It is a fundamental challenge in human health to understand disease mechanisms for diagnosis. In pharmacology and e-health research area, drugs play key roles to cure a disease and drug research focuses more on identification of the drug targets which can be manipulated to produce the desired effect with disease genes [2,3]. But, the therapies used nowadays against cancers are not effective and it is also extremely important to understand the underlying significant networks and mechanisms in order to prevent the progression of the tumor. Systems medicine is an emerging approach in systems biology that aims at integrating a large scale of the molecular interaction data and experimental data to elucidate diseases and drugs associations [4,5]. Therefore, identifying the potential set of drug targets to eliminate the disease genes and related networks is a valuable problem for drug design.

The traditional computational approaches utilize structural information to predict whether a protein can be a drug target [6]. Although these methods achieved reasonable performance, they suffer from limited availability of data such as protein 3D structures. Several algorithms have been proposed to predict drug-target associations by combining drug-drug and gene-gene similarity measurement [7-9]. Chemical structure of the drugs and sequence information of the target proteins were used to be the features to learn the classifier based on the target proteins as gold-standard positive dataset. However, the two dimensional chemical structure does not always comply with three-dimensional structural similarity [10]. Beside the sequence, structure-based approach, biological networks and functional annotations help us to understand the complexity of the cell. Bakheet et al. proposed a list of properties of human drug target proteins, including EC numbers, Gene Ontology (GO) term as features to predict potential drug targets [11]. However, GO terms include a brief description of the corresponding biological function of the genes but only 60% of all human genes have associated GO terms and these terms may be inconsistent due to differences in curators' judgment [12]. Based on the different features to predict the potential drug target must have the positive and negative data to build the accurate prediction model. But, it is technically difficult to determine negative data such like non-drug targets due to the lack of researchers who are interested in validating them. However, all of the above methods focus more on prediction the drug-target interactions which predict possible target proteins of drugs with unknown targets. Their approaches did not directly predict the potential protein which can be a drug target for a specific disease treatment.

The complex biological system is assembled and communicated with a set of interacting elements which are bound together by the interactions. Scale-free biological networks have a few nodes with a very large number of links (degree) and many nodes have only a few links [13]. This implies that the low-degree nodes may belong to the sub-networks which tend to form communities and those sub-networks are connected to each other through high degree nodes (hubs). It implies that the sparsely connected nodes in the network are part of highly clustered regions with communication and there is minimum number of steps from a given gene to the other genes. This event also denotes that a given genes can grow rapidly to the other communities by the hub connection which suffers from the small world effect [13]. Based on the characteristic of biological network, it provides a framework for understanding the pathophysiological phenomena and network-based analysis aims to harness this knowledge to understand the impact of intervention [14]. We explore computational strategies for identifying drug target, aiming to identify important nodes in the network. The bridge-elements of biological networks such as nodes with higher degree can be important targets of network-based drug discovery [14]. Hubs with higher degree nodes are the centre of networks only from the point of local network topology and thus their removal may disrupt a number of essential pathways to break cancer network. This method takes all the interaction into consideration but they do not further select active interaction relationships among proteins while only a part of the interactions among a set of proteins may be active at a specific condition [15]. Another strategy considers more global attribute such as closeness/betweenness centrality and a node with higher value of closeness/betweenness centrality would be interpreted as initial candidates for drug targets [16]. A common criticism of those global attributes that they are measured based on the shortest paths and they do not take into account non-shortest paths while spreading the information in the network. Those global measures recently have a variation based on network flow which has been proposed in [17]. On the other hand, given the set of the essential disease-related pathways and complexes, Hormozdiari applied the graph cut approaches to disrupt all the paths which can communicate with those essential pathways [18]. But we have limited or even no information about the real possible disease-related pathways.

Now, computational methods become increasingly attracting the attention of biologists who wish to identify the effective drug target. Although we do not known the real ideal routes in the biological network, gene expression will depend upon the mechanism as a consequence of action through regulatory adjustments as well as the changes it induces in the cell. Therefore, our idea is to gather the knowledge of the interaction networks and gene expression data to develop a network flow-based approach for potential drug target discovery which can produce strong effect on the disease genes.

## Methods

**Figure 1:**
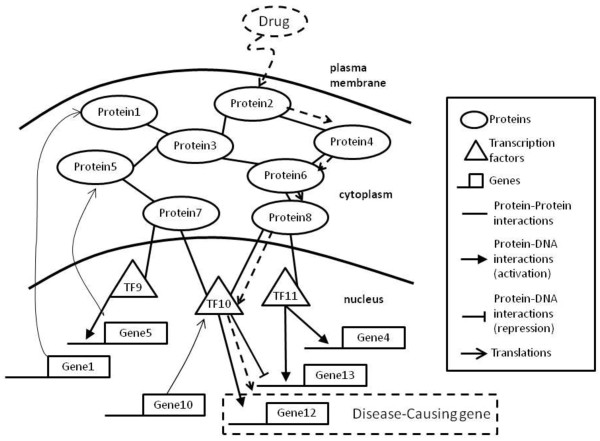
**the biological networks containing gene regulation and signal transduction **[20].

We first define our method as input a set of candidate proteins set C (known to be drug targets of the approved drugs treated in human), disease genes set D (known to be associated with the specific disease of interest). The mutations of the genes lead to increase or decrease in their gene expressions and the change of the protein functions and this information can help us to identify the new drug target for therapeutic intervention. Rapid identification of the drug targets needs to understand the underlying essential functional networks modulated by the transcription factors which may be affected by human diseases [19,20]. Take an example in Figure 1, gene 5 is regulated by transcription factor 9 in nucleus and translates to the protein 5 and protein 5 interacts with other proteins in cytoplasm. The dash line presents the possible paths after the drug treatment for disease gene 12. Subsequently, we integrate protein-protein and protein-DNA interactions and assign weight values of interactions based on the Pearson correlation and expression changes between experimental and control data. We present a network flow approach to infer the relationship between candidate proteins and disease genes. The aim of this problem is to rank the proteins in C based on the influenced effects of the disease genes D.

### Microarray data pre-processing

Gene expressions either over-expressed or under-expressed can be revealed in terms of two colored channel in the microarray data representing the intensity of the developmental stage such as cancer and normal samples. The gene expression ratios are calculated as the median value of the pixels minus background pixel median value for one color channel divided by the same for the other channel. We extract the median value of the log base 2 of each gene in experimental dataset because the median value of the normalized ratio is much harder to be affected by noise than the mean value. In order to get as complete data as possible, we use the K Nearest Neighbors (KNN) algorithm [21] to estimate the missing values. There are 11130 genes after removing genes that have more than 20% missing values in the microarray dataset.

### Edge weighted network construction from microarray data and public interaction databases

Protein-protein interaction (PPI) networks are the assembly of the protein signal cascades that transfer the biological function and information through the pathways [22]. Current public PPI databases provide rich information and they mostly differ on the way they acquire or validate their data. For example, HPRD, BIND, MINT and MIPS are manually curated, this means a team of biologists check the literature to find new interactions and once an interaction is confirmed it is added to the database. On the other hand, DIP and IntAct are based on literature mining and they achieve these using computational methods that retrieve the interaction knowledge automatically from published papers. Prieto and De Las Rivas have shown a limited intersection and overlap between the six major databases (BioGRID, BIND, MINT, HPRD, IntAct, DIP) [23]. The information contained in these databases is partly complementary and the knowledge of the protein interactions can be increased and improved by combining multiple databases. We integrate PPI data warehouse with those six major databases and erase the duplicated interaction pairs using the synonym of the protein name. We map the synonym of the protein name and then we erase the duplicated interaction pairs and successfully gather 54283 available and non-redundant PPI pairs among 10710 proteins [24]. Due to the limitation of the knowledge of the directed interactions between transcription factors and genes under specific condition, we gather the associated interactions under prostate cancer developed by Yeh et al. who used statistical assessments to construct directly regulatory associations between transcription factors and genes based on the gene expression data and transcription factor binding site prediction toolkit [25]. They have already constructed the relationships between transcription factors (PBX1, EP300, STAT6, SREBF1, NFKB1, STAT3, EGR1, E2F3, NR2F2) and their regulatory networks and validated by public literature and databases [25]. Finally, we collect 13363 directed edges and 20567 bi-direction edges as our network.

The human interactome network will be much more biologically insightful while the microarray expression data is taken into consideration. We assume that a protein interaction participates in a signaling pathway and the genes producing the associated proteins should be co-expressed and might be co-regulated. Grigoriev showed that biologically relevant interacting proteins have high mRNA expression correlations and the correlation of the expression genes provides some evidences and biological needs for the produced proteins in the co-expression network linked in PPI [26]. Co-expression networks are most commonly used for identifying cellular networks and the expressions of genes in co-expression networks are highly to form functional network modules [27]. Those networks are built on the expression profile similarity measure which is often calculated by Pearson correlation [27] and the correlation can measure the strength of a linear relationship between two expression values of the genes in the microarray data. Due to the drug target is treated in cancer network, we apply Pearson correlation coefficient for every pair-wise relation in the set of (N^2^-N)/2 pairs of N genes in cancer data and the range of the value would be [-1,+1]. In general statistical usage, the positive value in Pearson correlation indicates an increasing linear relationship and negative value indicate a decreasing linear relationship. While the correlation is closer to +1 or -1, it denotes the perfect linear relationship between pair of genes. On the other hand, the correlation approaches to zero and there would be little or no association among the pair of genes. We take the absolute value of correlation value to capture inhibitory activity (negative correlation) as well as activation activity (positive correlation). Therefore, we define the edge capacity in Equation (1) as the product of the absolute value of Pearson correlation *R_ij _*and the sum of the absolute value of differential expression changes between the normal and the cancer microarray data of the two corresponding genes i and j in the edge.

(1) $$w_{e}=w_{ij}=\left|R_{ij}\right|\times \left(\left|E_{ci}-E_{ni}\right|+\left|E_{cj}-E_{nj}\right|\right)$$

where *w_ij _*denotes the capacity function from edge e of node i to node j. |*R_ij_*| is the absolute value of Pearson correlation coefficient for the interaction of the node i and node j from cancer samples. The higher weight of pair of genes denotes the stronger correlation and differential expression exchanges between any two of the genes. One the other hand, the weight is zero indicates the two proteins are uncorrelated and no flow can pass through. *E_ci _*is the average gene expression value of node i in the cancer microarray data and *E_ni _*represents the average expression value in the normal microarray data.

### The network flow approach to discover the drug target

We gather 322 known drug targets treated in human extracted from DrugBank [28] database to be candidate proteins and 108 disease genes from curated database OMIM ID #176807 [29], KEGG pathway database with entry hsa05215 [30], PGDB database [31] as truly prostate cancer genes see Additional file 1. We identify our problem of discovering potential drug targets as a maximal flow problem that is to find out the amount of flows that can pass between candidate proteins C and the disease genes D in the networks. Maximizing the flow amount from the candidate proteins to the disease genes is reasonable because the efficiency in the biological networks can award a treatment advantage to the disease.

We make the following constraints to specify our problem into the classical maximum flow problem [32,33]:

(1) Constraints 1: The edge of a transcription regulator gene to its dependent genes is directed while a PPI is a bi-direction edge.

(2) Constraints 2: We create a dummy sink node which links to all possible disease-causing genes based on the public databases and the capacities on these edges are all set to infinite.

(3) Constraints 3: We create a dummy source node which links to one of the candidate proteins from the DrugBank database [28] and the capacities on these edges are set to infinite.

(4) Constraints 4: If a disease gene is also a candidate protein, we only link this gene to dummy source node and do not link to the dummy sink node. Because it is not fair to link the known disease genes to dummy sink nodes which have an infinite flow.

According to the above constraints, we define human interactome network as an edge-weighted graph G = (V, E, W), where vertex u, v belong to V-{s,t}, V is a node set of genes, s and t are source and sink nodes respectively and E is an edge set and each edge (u, v)∈ E, and W is a non-negative capacity on the edges calculated by Equation (1). This is different from the well-studied maximum-flow problem in which the capacity of each edge is a constant. The flow in a graph is dependent on the capacity of the edge and it must satisfy the following three conditions in order to find the maximum amount of flow from the source node to the sink node through the network [32,33]:

(1) Skew symmetry: f (u,v) = -f (v, u), for all u,v belong to V, f (u,v) is termed the flow from node u to node v. The property says that the reverse flow is the same amount but in reversed direction.

(2) Capacity constraint: c(u,v) > f (u,v), for all u,v belong to V-{s,t}, f (u,v) cannot exceed its edge capacity, so residual capacity is r(u,v) = c(u,v) - f (u,v), if r(u,v) = 0, then we call the edge (u,v) is saturated.

(3) Excess flow: Σ f (v,u) = excess(u) > 0 for all u, v belong to V-{s}, we say excess(u) is the summation of flows into the node u. We say that the node u is overflowing if excess(u) > 0.

**Figure 2:**
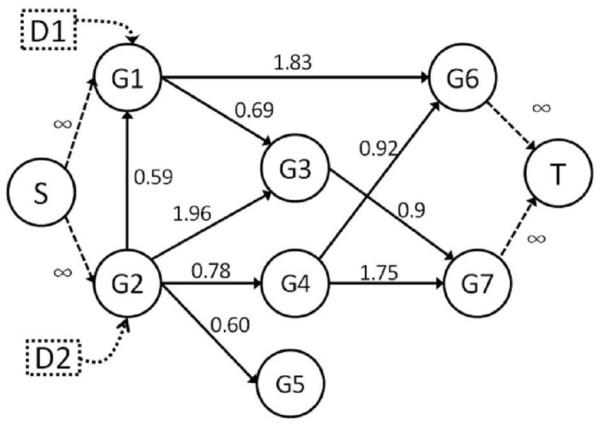
**Sample networks for the maximum flow and affected genes**.

We conduct the network flow approach to find the maximum flow between dummy source and sink nodes. First, we apply the shortest path algorithm [34] to calculate the distance between each node to the dummy sink node and set the shortest distance as the height of node. Take an example in Figure 2, the initial height of the node G1 is 2 because the shortest path between node G1 and dummy sink node T is 2. Second, the dummy source node starts to push the flow to its neighbors. While there exists a flow into the node u which denoted as excess(u) to be larger than zero, we can do "push" operation from node u to its neighbor node v based on the height label of the node. If the height of current node u is equal or less than the height of its neighbor node v, we set the height of node u as one more than the height of the neighbor node v (i.e. h(u) h(v) +1). Therefore, if the node u has excess flow and the height of a node equal or less than its neighbors (i.e. h(u)ᒭh(v)) while the residual capacity is greater than zero (i.e. r(u,v) > 0), we apply "relabel" operation to increase the height of the current node and push the flow from node u to node v. The node with positive excess flow is termed an active node and we select an active node and conduct the "push" and "relabel" operations to give the flow to dummy sink node repeatedly until there is no more active node. Finally, we run this procedure for each 322 candidate proteins and sort them based on the maximum flow. We supposed there be a probability density function of the maximum flow of the single candidate protein and there be parameters that can maximize the likelihood function to fit the density function. A simple and rapid method to calculate an approximate confidence interval is based on the application of the central limit theorem. We get the mean and variance to calculate the top 5% area as lower limits of the 95^th ^percentile confidence interval in the distribution of the maximum flow.

On the other hand, the inhibition of some proteins may also deactivate some non-disease genes and this might damage the normal functionalities of networks as undesirable side effects. In the procedure of the network flow approach, the flow may pass through the non-disease genes with different paths in the network and we simultaneously calculate those non-disease genes nodes using Equation (2) and (3) based on all the nodes retrieving a flow and their neighborhood nodes [35].

(2) $$P_{i}=F_{i}\;/\;K_{i}$$

(3) $$AG\left(D_{m}\right)=\underset{i=1}{\sum }P_{i}$$

where *P_i _*is the affected ratio of a node i in which *K_i _*is the total number of incoming neighbors of the node i and *F_i _*is the number of neighbors of node i which push the flow to node i. *AG(D_m_) *denotes the total affected genes of candidate proteins which is the sum of affected ratio *P_i_*'s of all the nodes while running the maximum flow procedure on the m^th ^candidate proteins.

We take an example to illustrate a small directed edge-weighted network using our method in Figure 2. We define gene G6 and G7 as disease genes and create a dummy sink node T to capture the flow from the gene G6 and G7. To illustrate, the infinite flow is coming from the G1, the flow between G1 and G3 limit to 0.69 due to the edge capacity. On the other hand, the edge capacity between G3 and G7 is 0.9 but the flow from G3 only have 0.69 can pass to node G7. The flow from G7 to T is by the same reason. The detailed procedure of our method is shown in Table 1. We calculate candidate protein G1 of drug D1 with the maximum flow 2.52 to both disease gene G6 and G7 using our method. This procedure denotes that if one of the edge capacities is small and it will limit the flow in the whole path. If there are more paths between candidate proteins and disease genes, the maximum flow may be larger. Since the number of incoming degrees of G1, G3, G6 and G7 are all 2 (K_i _= 2) and each node receives flow only from one of its incoming edges (F_i _= 1). For example, gene G3 has two incoming edges from node G1 and G2 but only gene G1 pushes flow to G3. So, we calculate each of the P_i _for G1, G3, G6 and G7 is 0.5 based on Equation (2) and AG(D1) = 2 based on Equation (3) respectively. For single candidate protein G2, we can similarly compute its maximum flow as 3.17 and the AG(D2) = 6. We note that the flow of candidate protein G2 is greater than that of gene G1, so drug D2 may be more effective than drug D1.

**Table 1:** The detailed procedure of our method on drug target G1

Step	Push node	flow	Received node
1	S	∞	G1
2	G1	1.83	G6
3	G1	0.69	G3
4	G3	0.69	G7
5	G6	1.83	T
6	G7	0.69	T

## Experiments

Prostate cancer is a frequently diagnosed as a hormone refractory and aggressive metastasis cancer and there is a pressing need for the development of new treatments. Prostate cancer frequently progresses from an androgen dependent disease but it can also transit to the androgen independent disease which is useless for taking androgen ablation therapy. We use prostate cancer as our test domain and integrate microarray data taken from [36] that consists of 62 primary tumors and 41 normal tissues from Stanford Microarray Database (SMD) [37]. DrugBank is the database that collects all FDA approved drugs and their targets and it contains 3516 drugs and 1046 drug targets. Most of the drugs have only one target and most genes are targeted by more than one drug. There are 16 overlapped genes between candidate proteins and truly prostate cancer genes and those genes only link to the dummy source node based on the constraint 4. We get 20 drug targets which belong to the "approved" and not "nutraceutical" drugs for prostate cancer in DrugBank and nine drug targets are also in the microarray data to be our tested benchmark. We implement our methods in java programming language with CPU Intel 1.73GHz and 1GB main memory running under the windows operating system.

### Single drug target discovery by maximum flow approach

**Figure 3:**
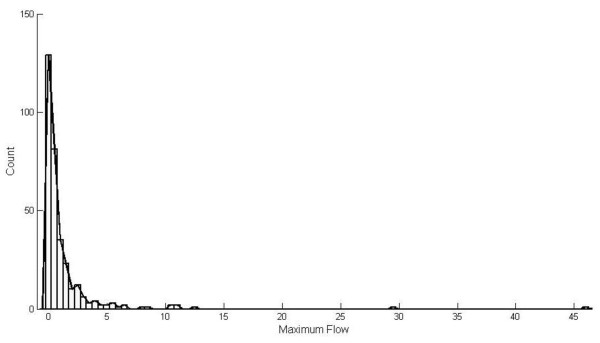
**the distribution of the maximum flow of single drug target**.

We show the distribution of the maximum flows obtained from the 322 candidate proteins in Figure 3 and the detail information of the maximum flow and affected genes of each candidate protein see Additional file 2. We display 22 candidate proteins at the top 5% maximum flow as lower limits of the 95^th ^percentile confidence interval in Table 2. The candidate protein with the highest maximum low has at least node degree 30 is 14/22 (64%) and as a result hubs may be also considered for further evaluation as being potential drug targets [38].

**Table 2:** Top 22 single drug targets for prostate cancer

Drug name	Target Name	F(Du)	AG(Du)
Casodex, Eulexin, Striant Bucl	AR	45.88	5576.87
Erlotinib	EGFR	29.54	4272.24
Budesonide	NR3C1	12.52	4677.47
Estramustine	ESR1	10.88	4597.53
5-[4-(1-Carboxymethyl-2-Oxo-Propylcarbamoyl)-Benzylsulfamoyl]-2-Hydroxy-Benzoic Acid	CASP3	10.84	3914.63
Glycerol	TGFBR2	10.40	2943.79
ATL1101	IGF1R	10.29	4080.81
Dodecane-Trimethylamine	PTPN11	8.43	3678.47
Fica	CASP7	7.80	3276.11
Diphenylacetic Acid	CTSB	6.62	3391.85
Marimastat	MMP2	6.29	3518.70
	SLC25A6	5.75	3994.49
	AURKA	5.60	3256.80
Phenelzine	MAOA	5.40	3067.25
	LGALS3BP	5.34	2902.46
	LCK	5.22	3607.57
	THBS1	5.10	2451.01
Insulin, porcine	INSR	4.32	2344.82
Trastuzumab	ERBB2	4.31	3351.47
Staurosporine	CDK2	4.00	3692.15
K-252a	MET	3.90	2318.86
	TFRC	3.83	2611.10

Although the biological validation for the predicated results from our method is difficult, it turns out that some of our predicted results had been reported in the public literature for validation. Our approaches find androgen receptor (AR) which has the rank 1 in our results and suitable for androgen-deprivation therapy in prostate cancer [39]. Casodex, Eulexin, Striant Bucl all use AR to be their drug targets. Erlotinib, a tyrosine kinase inhibitor targeted EGFR, is known to be over-expressed in prostate cancer and clinical showed that the inhibition of EGFR has implicit antitumor effects in prostate cancer [40,41]. CASP-3 and -7 are critical genes involved in cytokin and apoptosis interactions and they are also potential drug targets for cancer [42]. However, the drug target CASP3 is strongly related to several diseases and it is hard to use as a specific property to distinguish the prostate cancer. The membrane receptors and related genes of TGFB are associated with tumor suppressor functions for controlling cell apoptosis in the progression of prostate cancer and it is also related to promote disease progression of prostate cancer. Inhibition of TGFB has been shown to suppress the growth of androgen-independent tumors and it might be a valuable therapeutic strategy for androgen-independent disease [43]. Our approaches also identify gene IGF1R which is annotated as a best known family of cell signaling molecules and have already been cited in the clinical drug development [44]. Major pharmaceutical companies which focus on treatment of the hormone refractory prostate cancer also develop the drug antisense inhibitor ATL1101 with drug target IGF1R to treat the human cancer and it has already shown in laboratory studies with potential activity of inhibiting programmed cell death for prostate cancer treatment [45]. Most of the mutation in PTPN11 gene leads to the SHP2 variants and transits the signals of the mitogenic and pro-migratory via the activation of the RAS/ERK cascade. SHP2 is also the key target of the oncogenes which is also shown to affect the cell proliferation and neu-induced transformation in mouse modes and the inhibition of the SHP2 offers the potential new therapeutic avenues in the cancer [46]. Other researches and our method both suggested that Marimastat is a MMP2 inhibitor which may have effective biological activities for prostate cancer [47]. AURKA kinases are strongly expressed that are related to the wide range of the cancer types and their expressions are also associated with the gene amplification in tumor cell, genetic instability and poor prognosis [48]. AURKA is related to the G2-M transition in the cell cycle and the inhibition of this gene may lead to the G2-M arrest and apoptosis [48]. Inhibitor of monoamine oxidase A (MAOA) is commonly used to treat the neurological conditions and other researchers identified the higher expression MAOA in prostate cancer comparing with the normal cell [49]. Our findings suggest that the inhibitors of MAOA may be an anti-oncogenic therapy for prostate cancer and the results are consistency with the recent experimental results. The HER2/neu (ERBB2) oncogene plays an important role in hormone-related cancer associated with the prostate cancer and previous clinical studies showed that the effects of inhibitor of ERBB2 tyrosine kinase activity inhibit PC-3 cell growth [50]. The research reported that K252a with drug target MET inhibits the proliferation of the androgen-independent human prostate carcinoma cell line and it causes the change of the cell cycle [51]. There are 4 disease genes (AR, EGFR, ESR1, and ERBB2) to be potential drug targets in our top 22 candidate proteins of the 95^th ^percentile confidence interval and it show that the truly prostate cancer genes do not always direct to the drug targets.

**Figure 4:**
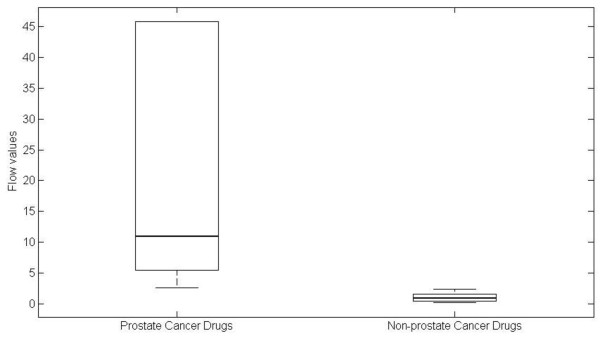
**the maximum flow between prostate cancer and non-prostate cancer**.

We define set of the truly drug targets which are annotated for prostate cancer drugs deposited in DrugBank database [28] and other drug targets are regarded as non-prostate cancers drug targets. Figure 4 shows the maximum flow between them and the results show our method can screen out potential drug targets for prostate cancers with higher maximum flow.

### The effect of the partially-directed and directed graph in our method

**Figure 5:**
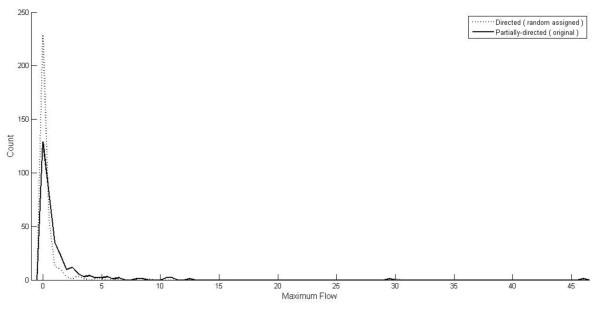
**the distribution of the maximum flow between partially directed and directed graph**.

**Figure 6:**
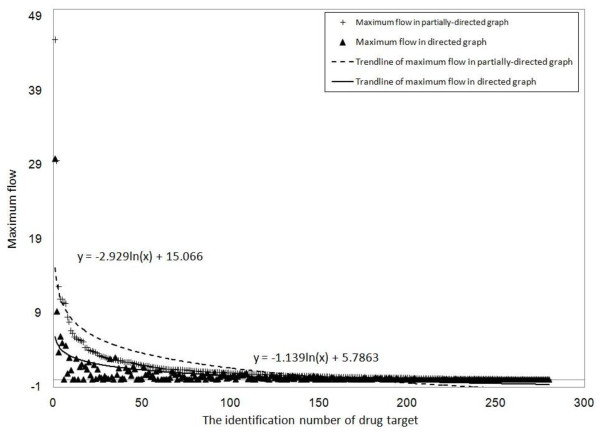
**the maximum flow of each drug target between partially directed and directed graph**.

The network we use is only partially directed graph and we conduct experiments to compare the performances of our methods against both partially directed and directed graphs. We randomly assign each undirected edge with a direction so that its corresponding partially directed graph can be transferred into a directed graph. Figure 5 shows the distributions of the maximum flow of candidate proteins between the original partially directed network and its corresponding transferred directed network. The similar trends in both graphs do not seem to affect the effectiveness of our approach using partially directed interaction data. Figure 6 denotes the regression to calculate the trend line for the maximum flow against candidate proteins while x-axis represents the identification number of the candidate proteins and y-axis represents the log of maximum flow. The fitting function of the overall maximum flow in partially-directed graph is y = -2.929ln(x)+15.066 and those in directed graph is y = -1.139ln(x)+5.7863. The distributions of those two graphs are similar but the partially directed graph tends to provide a much higher maximum flow for the disease genes. It implies that the flow can use different paths to reach the disease-related genes by passing the bi-direction edges in the partially directed graph while the directed graph may restrict the flow to reach the disease genes.

### The performance of our method

We use the same test data to show the performance between our method and the previous approaches such as degree, network entropy, betweenness, closeness and random walk. Degree centrality is defined as the number of links incident upon a node and rank genes based on their degree. Another network attribute called network entropy which is a characteristic measure of topology configuration based on the degree distribution [52] and this is consistent with the Shannon entropy of the distribution [53]. Closeness centrality is the normalized number of steps to access every other node from a given node in a network by calculating the geodesic distance of every node to every other node. Betweenness centrality denotes the fraction of the nodes occur in the shortest paths comparing with the other nodes. Otherwise, we apply the same scoring function as Equation (1) to assign the weight to the edges and calculated betweenness and closeness by JUNG toolkit [54]. Besides the closeness/betweenness centrality modeled by the use of shortest paths, Random walk capture more all type of communication scenarios and denotes the fraction of time spent 'visiting' the node through the edges in the network [55]. We use candidate proteins as start nodes to apply Random walk with alpha value 0.3 and sum up the probability of disease genes to rank the candidate proteins.

**Figure 7:**
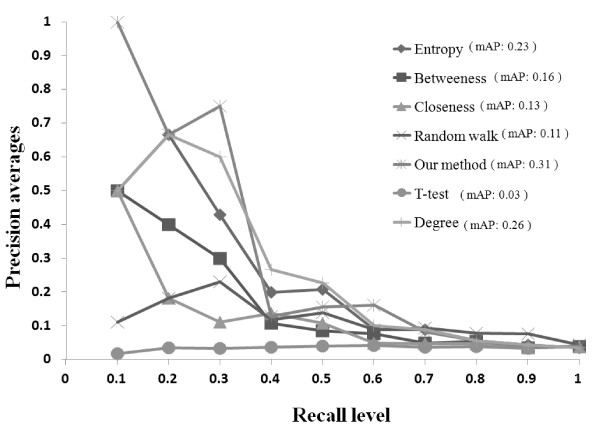
**the precision and recall curve among previous and our methods where mAP denotes is the mean of the average precision scores for each level recall level**.

Previous study showed that precision-recall curves give a more informative of an algorithm's performance based on the skewed degree-distributions of the scale-free biological network [56]. Precision is defined as the fraction of truly drug targets identified among the candidate proteins ranked above the particular position, whereas recall is defined as the fraction of truly drug targets identified among total number of the truly drug targets. We plot average precision vs. recall level curves by varying the rank of a candidate protein to be considered a truly drug target in Figure 7. We obtain the highest mean average precision as 0.31 and the results show that our method outperforms all other methods. The results based on the statistical t-test method denotes that only dependent on the gene expression values without network information supported is not suitable to discover the potential drug targets and genes to be true drug targets may have small differential expression changes. Unfortunately, it turns out that attributes of the network topology are not strongly correlated to the lethality of removing the associated protein from the network due to the noise in the interaction network [17].

In particular, we also compare these methods in terms of the average rank of the truly drug targets. Clearly, lower average rank indicates better performance. We report the percentage of truly drug target that are ranked in the top 1% of all candidate proteins (practically in the top 3 genes), in the top 3% (practically in the top 10 genes) and in the top 15% (practically in the top 48 genes) among all candidate. The absolute count and average position (AP) of found truly drug target is presented in Table 3. For instance, in the top 1%, there is a tie between degree and our method, where both find 2 truly drug targets. However, degree has AP of 2.5 which is larger than those of our method. In the top 15%, our method can find largest number of truly drug targets and also get lower AP than closeness and random walk method which get less number of truly targets.

**Table 3:** number of known prostate cancer drug targets and its average position among different methods in top 1%, 3% and 15% of the candidate proteins list

	Top 1%		Top 3%		Top 15%	
**Method**	**AP**	**Number**	**AP**	**Number**	**AP**	**Number**

t-test	N/A	0	N/A	0	N/A	0
Degree	2.5	2	3.33	3	9.4	5
Betweeness	2	1	5.67	3	13.5	4
Closeness	2	1	2	1	23	5
Random walk	2	1	9	1	20.6	5
Entropy	2.5	2	4	3	11.2	5
Our Method	2	2	2.67	3	18	6

## Conclusion

In this paper, we formulate the identification of drug target prediction as a maximum flow problem on biological networks. Previously, maximum flow approach is applied to do the graph cut which is popular related to computer vision research area and it separates the sources and targets in the network. To our knowledge, there are no computational methods that use the idea of maximum flow to predict potential drug target for specific disease treatment before this work. We successfully identify potential drug targets which are strongly related to the existing well known drugs for prostate cancer treatment and also discover more potential drug targets that could attract the attention to biologists. Our methods do not know the real ideal routes in the disease network but try to find the feasible flow to give a strong influence to the disease genes through possible paths using maximum flow approach. A significant difference between our method and random walk is the way that we treat the length of the path searched in the network. Random walk is more sensitive to the length of the paths with its matrix multiplication. The reason why we exclude the metabolic pathways in the networks is that it is hard to measure the whole kinetic parameters in a metabolic pathway towards disrupting the function. However, it is possible to extend our network between proteins and chemical compounds to representation of metabolic pathways.

## Competing interests

The authors declare that they have no competing interests.

## Authors' contributions

SH participated in algorithm design, performed program and statistical analysis. HY carried out the design of the workflow, algorithm and molecular studies and drafted the manuscript. VW participated in its overall design and coordination of the research and helped to draft the manuscript. All authors read and approved the final manuscript.

## Supplementary Material

Additional file 1 **List of the prostate cancer genes**. We use 108 genes from OMIM, KEGG pathway database, PGDB database as the truly prostate cancer related genes. Click here for file

Additional file 2 **The maximum flow and affected genes of 322 candidate proteins**. The maximum flow and affected genes of 322 candidate proteins. Click here for file
