# Supplementary material for: A network flow approach to predict drug targets from microarray data, disease genes and interactome network - case study on prostate cancer
Source: J Clin Bioinforma. 2012 Jan 13;2:1. doi: 10.1186/2043-9113-2-1 (PMC3285036; doi:10.1186/2043-9113-2-1)
Supplement: Additional file 1 — List of the prostate cancer genes. We use 108 genes from OMIM, KEGG pathway database, PGDB database as the truly prostate cancer related genes. [file 2043-9113-2-1-S1.PDF]

Addition files 1 : List of the prostate cancer-related genes

EIF3S3	CAV1	TYR
BCL2	CDKN1A	NCOA3
TCEB1	CDH13	KLK10
PLAU	ESR2	NAT1
MYC	EDNRB	IL8
KLK2	FHIT	TGIF
AR	NCOA4	KLK3
SLC2A2	DAPK1	GSTT1
IL12A	RARB	CYP1B1
HIF1A	RASSF1	VEGF
SOX2	GSTP1	TNF
EGFR	STMN1	PGK1
CCND1	PGR	TCF2
ERBB2	NKX3-1	EHBP1
CDH1	HRAS	ATBF1
ERCC5	BTRC	PLXNB1
FAS	IGFBP3	NEFL
TP53	SMARCA42	MXI1
PTEN	COPG	VDR
MAP2K4	ARMET	ELAC2
ANXA7	RNF14	EPHB2
CDKN2A	BRCA2	HIP1
ABL1	CHEK2	KLF6
SYNPO2	HOXA13	RNASEL
BRCA1	SMAD2	ESR1
CTNNA1	NME1	CD44
CTCF	CYP2D6	CDKN1B
TUSC3	PTPN13	BMP6
CD82	MAD1L1	GGT1
RB1	MSR1	FAF1
POLB	CYP3A4	APC
EGR1	TMPRSS24	MME
CTNNB1	FOLH1	STEAP3
MSH2	SYT7	SLC43A12
ATM	APRIN	CYP17A12
ACPP	TMEPAI	MC1R
