# Supplementary material for: A network flow approach to predict drug targets from microarray data, disease genes and interactome network - case study on prostate cancer
Source: J Clin Bioinforma. 2012 Jan 13;2:1. doi: 10.1186/2043-9113-2-1 (PMC3285036; doi:10.1186/2043-9113-2-1)
Supplement: Additional file 2 — The maximum flow and affected genes of 322 candidate proteins. The maximum flow and affected genes of 322 candidate proteins. [file 2043-9113-2-1-S2.PDF]

**Addition files 2 :** The maximum flow and affected genes of drug targets

| No. | DrugTarget | maximum _Flow | Affected genes |
|---|---|---|---|
| 1 | AR | 45.8781649 | 5576.868 |
| 2 | EGFR | 29.5355447 | 4272.238 |
| 3 | NR3C1 | 12.51828947 | 4677.473 |
| 4 | ESR1 | 10.8835855 | 4597.534 |
| 5 | CASP3 | 10.8420844 | 4152.823 |
| 6 | TGFBR2 | 10.39626266 | 2943.79497 |
| 7 | IGF1R | 10.2853364 | 4080.807 |
| 8 | PTPN11 | 8.431316696 | 3678.47354 |
| 9 | CASP7 | 7.799845335 | 3276.11001 |
| 10 | CTSB | 6.61534418 | 3391.845 |
| 11 | MMP2 | 6.286149674 | 3518.69656 |
| 12 | SLC25A6 | 5.751199083 | 3994.49165 |
| 13 | AURKA | 5.596951456 | 3256.80439 |
| 14 | MAOA | 5.398796348 | 3067.25297 |
| 15 | LGALS3BP | 5.343874199 | 2902.45615 |
| 16 | LCK | 5.224729925 | 3607.57237 |
| 17 | THBS1 | 5.097979788 | 2451.01338 |
| 18 | INSR | 4.317648711 | 2344.81614 |
| 19 | ERBB2 | 4.314003093 | 3351.47255 |
| 20 | CDK2 | 3.999061161 | 3692.15284 |
| 21 | MET | 3.898102231 | 2318.85947 |
| 22 | TFRC | 3.826589589 | 2611.10454 |
| 23 | ABL1 | 3.786449361 | 3168.72982 |
| 24 | RHOA | 3.683847958 | 2413.07279 |
| 25 | ACVR1 | 3.487459278 | 1940.24413 |
| 26 | PTK2 | 3.288461529 | 2165.80298 |
| 27 | RAF1 | 3.148799648 | 2490.97401 |
| 28 | SYK | 3.099073773 | 3108.22696 |
| 29 | PCAF | 3.035665509 | 3169.83219 |
| 30 | PTPN1 | 2.95417062 | 1880.07978 |
| 31 | TOP2A | 2.8370392 | 2183.0556 |
| 32 | BCL2 | 2.764359513 | 2148.28078 |
| 33 | F2 | 2.730460517 | 2041.60667 |
| 34 | SLC25A5 | 2.714487168 | 2671.40097 |
| 35 | ACACA | 2.704359896 | 1913.6313 |
| 36 | GSTP1 | 2.613759483 | 2457.02362 |
| 37 | ANXA1 | 2.592443849 | 1970.95116 |
| 38 | STS | 2.588698479 | 2650.49594 |
| 39 | PLAU | 2.521643353 | 2478.50062 |
| 40 | EDNRB | 2.372729129 | 614.782732 |
| 41 | CASP1 | 2.370569699 | 1750.82604 |
| 42 | BRAF | 2.363525883 | 1952.41629 |
| 43 | BIRC4 | 2.363525883 | 1952.41629 |
| 44 | MAPK14 | 2.319193602 | 3730.81944 |
| 45 | IFNAR1 | 2.222853672 | 2603.53694 |
| 46 | AKR1B1 | 2.154027585 | 1995.12098 |
| 47 | OAT | 2.105203661 | 1480.97326 |
| 48 | SPARC | 2.04792393 | 1874.81319 |
| 49 | SHMT2 | 2.01512007 | 2166.39134 |
| 50 | NME1 | 1.91207993 | 1008.02161 |
| 51 | CD59 | 1.910564818 | 1472.92299 |
| 52 | COL1A1 | 1.888902878 | 1502.4422 |
| 53 | MDM2 | 1.824287514 | 2993.22246 |
| 54 | C1R | 1.820169172 | 1383.88518 |
| 55 | KIT | 1.714103433 | 1883.22527 |
| 56 | LGALS3 | 1.69310626 | 1664.63111 |
| 57 | PLAUR | 1.647814589 | 2032.94066 |
| 58 | IL1R1 | 1.633680707 | 2707.11684 |
| 59 | SLC25A4 | 1.600134071 | 1106.75503 |
| 60 | CSK | 1.560437414 | 1231.98947 |
| 61 | VEGFA | 1.52114149 | 1648.01709 |
| 62 | SNRPA | 1.50773057 | 1898.60761 |
| 63 | SLC6A8 | 1.442316033 | 1032.65424 |
| 64 | RARA | 1.441332661 | 2628.09241 |
| 65 | TF | 1.416068811 | 2092.90967 |
| 66 | FGFR2 | 1.387010245 | 2374.98274 |
| 67 | CTBP1 | 1.377550591 | 2639.34129 |
| 68 | IFNGR1 | 1.37547834 | 1299.1078 |

| | | | | | | | |
|---|---|---|---|---|---|---|---|
| 69 | CASK | 1.37428494 | 1940.42739 | 106 | CSF2RB | 0.825739627 | 1992.59054 |
| 70 | XRCC4 | 1.357167362 | 1133.52118 | 107 | ANTXR2 | 0.817927417 | 672.299041 |
| 71 | ARAF | 1.355272888 | 1204.24586 | 108 | COMT | 0.787389499 | 1223.55363 |
| 72 | PTGS2 | 1.33243181 | 100.842938 | 109 | GHR | 0.764937411 | 1816.25023 |
| 73 | GOT2 | 1.327707581 | 1494.41779 | 110 | DDX6 | 0.763419895 | 1243.92627 |
| 74 | FGA | 1.311765031 | 1699.00101 | 111 | PIM1 | 0.759040199 | 1754.53724 |
| 75 | SNAP25 | 1.301988753 | 1598.99142 | 112 | MAPKAPK2 | 0.758792737 | 1492.31826 |
| 76 | POLA2 | 1.265645525 | 2665.7155 | 113 | TAP1 | 0.74851361 | 1139.39059 |
| 77 | PDE4D | 1.264539784 | 1313.71259 | 114 | PLA2G2A | 0.745307053 | 1229.94812 |
| 78 | F5 | 1.222524016 | 936.980176 | 115 | HTR2A | 0.742463815 | 1498.53452 |
| 79 | ABCA1 | 1.186291438 | 1418.9995 | 116 | PRLR | 0.737826423 | 1735.28187 |
| 80 | RARB | 1.181216087 | 2152.14545 | 117 | IFNGR2 | 0.736905374 | 995.444292 |
| 81 | THRA | 1.151482104 | 1310.88218 | 118 | PDE5A | 0.731934172 | 962.736297 |
| 82 | PRKAA1 | 1.13330491 | 1605.01525 | 119 | IGHG1 | 0.708993562 | 1143.18755 |
| 83 | COPG | 1.114320038 | 1724.15233 | 120 | EDNRA | 0.67735693 | 37.0889759 |
| 84 | ITGA4 | 1.112138224 | 1000.46125 | 121 | PITPNA | 0.661112173 | 860.358981 |
| 85 | FKBP4 | 1.109451474 | 1545.93271 | 122 | TNF | 0.654718961 | 1043.65813 |
| 86 | TUBB | 1.068729195 | 2489.98231 | 123 | CAT | 0.632047061 | 112.36286 |
| 87 | CTLA4 | 1.065259507 | 792.880965 | 124 | CD46 | 0.621921996 | 793.084028 |
| 88 | MAP2K1 | 1.019321268 | 1381.937 | 125 | FTL | 0.608094268 | 864.637364 |
| 89 | PARP1 | 1.014177742 | 2347.58587 | 126 | CACNA1C | 0.607765445 | 1673.28805 |
| 90 | PLAT | 1.01261485 | 1213.12004 | 127 | NP | 0.601214729 | 1291.9363 |
| 91 | ACAT2 | 1.006146564 | 1826.2059 | 128 | ATP1A1 | 0.591789101 | 818.602183 |
| 92 | IMPDH2 | 0.989455129 | 1592.86975 | 129 | F8 | 0.573324453 | 710.765605 |
| 93 | PPP1CC | 0.988399995 | 1514.46748 | 130 | MTHFD2 | 0.559344271 | 1193.13881 |
| 94 | HGF | 0.977172557 | 1299.90246 | 131 | LTA4H | 0.55806174 | 1120.20611 |
| 95 | CD3E | 0.975161797 | 881.284625 | 132 | RAP2A | 0.551015874 | 983.403243 |
| 96 | DCI | 0.952237036 | 1234.84563 | 133 | GLUL | 0.548884316 | 1436.35848 |
| 97 | ERBB3 | 0.938182372 | 1307.06611 | 134 | FRAP1 | 0.546743344 | 951.069259 |
| 98 | RPS6KA5 | 0.934736358 | 3114.32656 | 135 | F13A1 | 0.542503844 | 959.939516 |
| 99 | RRM1 | 0.914399589 | 1265.72536 | 136 | TK1 | 0.537214661 | 687.482357 |
| 100 | BACE1 | 0.889279152 | 1481.1683 | 137 | CP | 0.520059511 | 927.891541 |
| 101 | DUT | 0.876932483 | 1266.04453 | 138 | APRT | 0.519461832 | 1172.10371 |
| 102 | ATIC | 0.867202659 | 1642.08711 | 139 | EPRS | 0.508386894 | 1410.79771 |
| 103 | GSTM2 | 0.857654325 | 1281.87609 | 140 | SERPINC1 | 0.505964784 | 408.998345 |
| 104 | CDK7 | 0.852101316 | 1033.16216 | 141 | TNK2 | 0.497318855 | 907.582232 |
| 105 | AHCY | 0.827217371 | 1310.34126 | 142 | ASS1 | 0.489465838 | 645.186509 |

| | | | | | | | |
|---|---|---|---|---|---|---|---|
| 143 | TUBB2C | 0.484942125 | 862.765852 | 180 | CTSS | 0.282040637 | 673.128215 |
| 144 | EEA1 | 0.480544149 | 853.852695 | 181 | PYCR2 | 0.280866617 | 953.886222 |
| 145 | HNF4G | 0.478277421 | 829.370566 | 182 | ACHE | 0.269181324 | 532.092123 |
| 146 | GPHN | 0.475429122 | 1021.37383 | 183 | ADORA1 | 0.269062236 | 621.678144 |
| 147 | CRYZ | 0.474740979 | 1056.11868 | 184 | IL6R | 0.26723936 | 1173.37498 |
| 148 | CHKA | 0.469405728 | 924.292782 | 185 | ATOX1 | 0.2662024 | 165.700727 |
| 149 | ARF4 | 0.464115004 | 497.086902 | 186 | UCK2 | 0.263091374 | 689.591873 |
| 150 | TLR2 | 0.461390363 | 1172.47244 | 187 | ACPP | 0.260924795 | 201.11165 |
| 151 | APAF1 | 0.45143932 | 590.026694 | 188 | HRH1 | 0.259263049 | 49.3895916 |
| 152 | HLA-DRB1 | 0.446416405 | 703.786452 | 189 | ALAS1 | 0.258796236 | 668.6226 |
| 153 | CLPP | 0.439749138 | 683.027861 | 190 | APCS | 0.25771971 | 731.275805 |
| 154 | ATP5D | 0.431325378 | 540.587924 | 191 | CD55 | 0.257354264 | 281.232082 |
| 155 | SERPINE1 | 0.413388504 | 593.907296 | 192 | SLC16A1 | 0.252272304 | 1025.27066 |
| 156 | ME2 | 0.40391996 | 1741.64306 | 193 | TARS | 0.251488589 | 978.604493 |
| 157 | ADRA2A | 0.389978943 | 628.013694 | 194 | NQO2 | 0.249108524 | 828.452167 |
| 158 | DAPK1 | 0.383669039 | 630.779509 | 195 | CRABP2 | 0.247629998 | 239.576086 |
| 159 | SEC11A | 0.368582573 | 855.177202 | 196 | METAP2 | 0.245629582 | 497.139087 |
| 160 | GPI | 0.367016852 | 115.644871 | 197 | LDHA | 0.240105101 | 685.66082 |
| 161 | HMGCR | 0.364440986 | 1278.09548 | 198 | HK1 | 0.235465404 | 462.863234 |
| 162 | NQO1 | 0.358309186 | 692.457085 | 199 | HADH | 0.231208751 | 706.027688 |
| 163 | FKBP1A | 0.356742636 | 654.730456 | 200 | LCMT1 | 0.230592276 | 815.879883 |
| 164 | YARS | 0.339319939 | 664.268231 | 201 | SHMT1 | 0.227821319 | 1324.17683 |
| 165 | DHPS | 0.338204927 | 718.925398 | 202 | GAD1 | 0.219784903 | 651.951905 |
| 166 | NR3C2 | 0.33723255 | 581.775173 | 203 | GLS | 0.219261139 | 354.064318 |
| 167 | AGTR1 | 0.332892841 | 1338.53067 | 204 | GMPS | 0.1990332 | 629.881438 |
| 168 | ACVR1B | 0.316942908 | 347.767903 | 205 | KCNJ2 | 0.198183194 | 644.00527 |
| 169 | CBS | 0.311929239 | 497.694436 | 206 | GABBR1 | 0.186893161 | 582.878396 |
| 170 | FARSA | 0.311665957 | 335.79427 | 207 | PRSS1 | 0.184540813 | 610.352611 |
| 171 | NARS | 0.311662012 | 757.200301 | 208 | SLC16A2 | 0.179551228 | 152.990075 |
| 172 | SLC16A5 | 0.311109818 | 502.983085 | 209 | UGCG | 0.176227711 | 366.349979 |
| 173 | KARS | 0.310256763 | 954.613626 | 210 | LARS | 0.172223273 | 1049.40621 |
| 174 | TBCA | 0.309781509 | 780.762126 | 211 | SERPINA1 | 0.169224706 | 610.352611 |
| 175 | ALDOA | 0.305331019 | 830.95526 | 212 | MTHFD1 | 0.163964167 | 529.935491 |
| 176 | CAMLG | 0.303118504 | 696.863869 | 213 | GRIK2 | 0.159499468 | 326.871459 |
| 177 | LDHB | 0.302771418 | 648.362291 | 214 | PRKAB2 | 0.156175597 | 880.061945 |
| 178 | ITGAL | 0.297368276 | 353.828541 | 215 | SLC25A13 | 0.156145451 | 125.975369 |
| 179 | SLC12A2 | 0.282450824 | 754.524734 | 216 | HTR2B | 0.153115511 | 413.739786 |

| | | | |
|---|---|---|---|
| 217 | RARG | 0.149264354 | 321.77698 |
| 218 | DOT1L | 0.148784472 | 243.867097 |
| 219 | GGCX | 0.147117347 | 259.301881 |
| 220 | HINT1 | 0.139682863 | 181.101171 |
| 221 | CCL5 | 0.139298359 | 317.352098 |
| 222 | CD52 | 0.136693641 | 104.975386 |
| 223 | P4HA1 | 0.135785891 | 186.450495 |
| 224 | PCYT1A | 0.133369806 | 153.776894 |
| 225 | TYMS | 0.129324288 | 227.815658 |
| 226 | PDE3B | 0.127168651 | 279.134811 |
| 227 | ADRBK2 | 0.126466893 | 443.439597 |
| 228 | GNPDA1 | 0.124714429 | 444.12746 |
| 229 | LIG3 | 0.122690686 | 96.3515582 |
| 230 | FHIT | 0.115953654 | 72.9990298 |
| 231 | RAB9A | 0.115228742 | 276.650281 |
| 232 | ABCC8 | 0.109085155 | 394.203559 |
| 233 | RFK | 0.108265727 | 414.264749 |
| 234 | PAPSS1 | 0.107676512 | 315.567315 |
| 235 | FCER1G | 0.105021226 | 192.048668 |
| 236 | SLC23A2 | 0.103039052 | 163.480812 |
| 237 | LDLR | 0.10231846 | 146.141997 |
| 238 | GOT1 | 0.097766729 | 123.84746 |
| 239 | HIF1AN | 0.087699991 | 225.583598 |
| 240 | ODC1 | 0.083955949 | 242.762611 |
| 241 | KCNK1 | 0.0787014 | 60.6152203 |
| 242 | SRM | 0.074834218 | 368.23869 |
| 243 | DCK | 0.069487259 | 76.714015 |
| 244 | GNRHR | 0.065216945 | 267.449629 |
| 245 | HEXB | 0.065041469 | 145.223256 |
| 246 | GLO1 | 0.062573518 | 328.358044 |
| 247 | SUCLG2 | 0.060803327 | 240.736029 |
| 248 | GCK | 0.060755817 | 751.309377 |
| 249 | KCNH2 | 0.059530598 | 165.766221 |
| 250 | GSR | 0.056470875 | 682.726355 |
| 251 | FDPS | 0.055579725 | 308.35431 |
| 252 | TRAPPC3 | 0.055304441 | 67.2977972 |
| 253 | HSD17B4 | 0.046667777 | 185.603772 |

| | | | |
|---|---|---|---|
| 254 | CTH | 0.045332041 | 166.655839 |
| 255 | SGPL1 | 0.044670154 | 183.785862 |
| 256 | OAZ1 | 0.043324902 | 167.46982 |
| 257 | FAP | 0.04276119 | 47.5109464 |
| 258 | SLC25A20 | 0.037925801 | 392.028236 |
| 259 | ATP2C1 | 0.034583744 | 53.0983185 |
| 260 | PLOD1 | 0.03421093 | 92.1386414 |
| 261 | ADH5 | 0.034006952 | 26.3278847 |
| 262 | PRDX2 | 0.03393816 | 17.3778431 |
| 263 | CKMT2 | 0.033183551 | 89.0587069 |
| 264 | PRDX5 | 0.033004927 | 335.406736 |
| 265 | HBB | 0.031538091 | 32.0197298 |
| 266 | RNASE1 | 0.031111302 | 18.5460207 |
| 267 | TGM3 | 0.025131657 | 28.4181309 |
| 268 | PISD | 0.024571327 | 90.7435843 |
| 269 | PPIF | 0.022766707 | 72.1648942 |
| 270 | CFD | 0.020468344 | 197.845667 |
| 271 | NT5C2 | 0.019780528 | 146.517185 |
| 272 | GM2A | 0.018683663 | 25.1021282 |
| 273 | CA12 | 0.018365675 | 505.718201 |
| 274 | PCCB | 0.017612087 | 30.0096806 |
| 275 | PCCA | 0.017612087 | 30.0874583 |
| 276 | ASL | 0.014509603 | 150.376632 |
| 277 | SLC7A11 | 0.007496674 | 37.5434361 |
| 278 | RBP2 | 0.004427633 | 62.2952328 |
| 279 | AGXT | 0.003228726 | 24.2284509 |
| 280 | MTAP | 8.60E-04 | 150.885196 |
| 281 | MGP | 0 | 0.25 |
| 282 | RNASE4 | 0 | 0.25 |
| 283 | UAP1 | 0 | 0.28571429 |
| 284 | YARS2 | 0 | 1 |
| 285 | SRR | 0 | 1 |
| 286 | AMD1 | 0 | 0.5 |
| 287 | GLDC | 0 | 1 |
| 288 | DECR1 | 0 | 1 |
| 289 | AOC3 | 0 | 0.33333333 |
| 290 | PROCR | 0 | 0.5 |

| | | | |
|---|---|---|---|
| 291 | CPM | 0 | 0.5 |
| 292 | QDPR | 0 | 0.66666667 |
| 293 | HMGCL | 0 | 0.75 |
| 294 | IMPA1 | 0 | 0.33333333 |
| 295 | CES1 | 0 | 1 |
| 296 | GART | 0 | 1 |
| 297 | NPR3 | 0 | 1 |
| 298 | ACSL3 | 0 | 0.5 |
| 299 | GALE | 0 | 0.66666667 |
| 300 | GLA | 0 | 1 |
| 301 | GSTZ1 | 0 | 0.66666667 |
| 302 | CFB | 0 | 0.28571429 |
| 303 | IVD | 0 | 0.66666667 |
| 304 | FECH | 0 | 1.1 |
| 305 | ASPA | 0 | 0.5 |
| 306 | ARG2 | 0 | 0.25 |

| | | | |
|---|---|---|---|
| 307 | BCAT2 | 0 | 0.5 |
| 308 | HAGH | 0 | 0.5 |
| 309 | AKR1C2 | 0 | 1 |
| 310 | KIF1A | 0 | 0.4 |
| 311 | ACSS2 | 0 | 0.5 |
| 312 | HSD17B1 | 0 | 0.4 |
| 313 | PDXP | 0 | 1 |
| 314 | MMAB | 0 | 0.25 |
| 315 | RBP3 | 0 | 0.25 |
| 316 | ALDH1A1 | 0 | 0.5 |
| 317 | FDX1 | 0 | 0.5 |
| 318 | ALAD | 0 | 1 |
| 319 | MUT | 0 | 0.5 |
| 320 | UROD | 0 | 1 |
| 321 | HMOX1 | 0 | 0.5 |
| 322 | ACAD8 | 0 | 0.66666667 |
